# Supplementary material for: Innovative approaches in soil carbon sequestration modelling for better prediction with limited data
Source: Sci Rep. 2024 Feb 8;14:3191. doi: 10.1038/s41598-024-53516-z (PMC10850547; doi:10.1038/s41598-024-53516-z)
Supplement: Supplementary file 1 — Supplementary Information. [file 41598_2024_53516_MOESM1_ESM.pdf]

Innovative Approaches in Soil Carbon Sequestration Modelling for Better Prediction with Limited Data

Mohammad Javad Davoudabadi[1,2,3,4,*], Daniel Pagendam[4], Christopher Drovandi[1,2,3], Jeff Baldock[5], and Gentry White[1,2,3]

[1] *School of Mathematical Sciences, Queensland University of Technology, Australia;*
[2] *Australian Research Council Centre of Excellence for Mathematical & Statistical Frontiers (ACEMS);*
[3] *QUT Centre for Data Science, Queensland University of Technology, Australia;*
[4] *CSIRO Data61, GPO Box 2583, Brisbane, QLD 4001, Australia;*
[5] *CSIRO Agriculture & Food, Glen Osmond, South Australia, Australia;*
[*] *mohammadjavad.davoudabadi@hdr.qut.edu.au*

Supplementary Material

A Notation

The notations related to latent variables $\mathbf{X}$ at time t and field i, their corresponding measured values $\mathbf{Y}$ and some model parameters are presented in Table S1.

All processes and all observations at time t in all fields (soil types) are denoted by $X_{(t)} = (X^1_{(t)}, X^2_{(t)}, X^3_{(t)})$ and $Y_{(t)} = (Y^1_{(t)}, Y^2_{(t)}, Y^3_{(t)})$, respectively. All processes at all fields (soil types) and all times are represented by $\mathbf{X}$, and $\mathbf{Y}$ represents all available data. We denote a set of variables as $Y_{1:t} = (Y_{(1)}, ..., Y_{(t)})$. The log-normal distribution is denoted by $LN(\mu_1, \sigma_1^2)$ with mean parameter μ_1 and variance parameter σ_1^2 for a log transformation of the random variable. $N(\mu_2, \sigma_2^2)$ represents the normal distribution with mean and variance μ_2 and σ_2^2, respectively. It is important to note that in these expressions, the subscripts '1' and '2' in σ_1^2 and σ_2^2 are placeholders. In practical applications, they will be substituted with abbreviations that correspond to specific pools or models being referred to, for clearer

Notation	Description
$X^i_{C(t)}$	The mass of SOC (t/ha)
$X^i_{W(t)}$	The mass of total wheat dry matter (t/ha)
$X^i_{S(t)}$	The mass of total sorghum dry matter (t/ha)
$X^i_{G_W(t)}$	The mass of total grain dry matter produced from wheat (t/ha)
$X^i_{G_S(t)}$	The mass of total grain dry matter produced from sorghum (t/ha)
$X^i_{P(t)}$	The mass of total pasture dry matter (t/ha)
$X^i_{IOM(t)}$	The mass of IOM (t/ha)
$X^i_{B(t)}$	The mass of BIO (t/ha)
$X^i_{D(t)}$	The mass of DPM (t/ha)
$X^i_{R(t)}$	The mass of resistant plant material (RPM) (t/ha)
$X^i_{H(t)}$	The mass of HUM (t/ha)
$Y^i_{TOC(t)}$	The measured value of total SOC (t/ha)
$Y^i_{W(t)}$	The measured value of total wheat dry matter (t/ha)
$Y^i_{S(t)}$	The measured value of total sorghum dry matter (t/ha)
$Y^i_{G_W(t)}$	The measured value of total wheat grain dry matter (t/ha)
$Y^i_{G_S(t)}$	The measured value of total sorghum grain dry matter (t/ha)
$Y^i_{P(t)}$	The measured value of total pasture dry matter (t/ha)
$Y^i_{IOM(t)}$	The measured value of IOM (t/ha)
$Y^i_{H(t)}$	The measured value of HUM (t/ha)
$Y^i_{POC(t)}$	The measured value of POC (t/ha)
K_C	The decay rate of total SOC (Y^{-1})
K_A	The decay rate of the carbon in pool A (Y^{-1})
π_{AB}	Proportion of the mass of carbon transfer from carbon pool A to carbon pool B
Δt	The yearly time step
P_D	Proportion of the carbon input that added to the DPM pool

Table S1: The notations of latent variables, their corresponding measured values and some model parameters.

identification and differentiation. It is the same for subscripts 'A' and 'B' in K_A and π_{AB} in Table S1. Some other notations are presented wherever they are required.

B Prior and Proposal Distributions

The model parameters and their prior probability density functions related to the Tarlee and Brigalow datasets are listed in Tables S2 and S3. To avoid repetition, the priors of the model parameters which have the same distribution in both datasets are presented in Table S2. Given the variety of models and their respective submodels presented in this study, we advise readers to refer to Sections D through G. These sections provide detailed information on which parameters are associated with each specific model and submodel.

Parameter	Prior	Type
$X^1_{C(1978)}$	Truncated-normal$(40, 10^2, lower = 0)$	Uninformative
$X^2_{C(1978)}$	Truncated-normal$(40, 10^2, lower = 0)$	Uninformative
$X^3_{C(1978)}$	Truncated-normal$(40, 10^2, lower = 0)$	Uninformative
X_{IOM}	Truncated-normal$(4, 0.5^2, lower = 0)$	Uninformative
K_C	LN$(-2.71, (0.127)^2)$	Informative
K_D	LN$(-2.71, (0.127)^2)$	Informative
K_B	Uniform$(0, 1)$	Uninformative
K_R	LN$(-2.5, (0.135)^2)$	Informative
K_H	Truncated-normal$(0.02, 0.01^2, lower = 0)$	Informative
c	N$(0.45, (0.01)^2)$	Informative
r_W	N$(0.5, (0.067)^2)$	Informative
r_P	N$(1, (0.125)^2)$	Informative
p	Beta$(89.9, 809.1)$	Informative
h_W	LN$(0.825, (0.36)^2)$	Weakly Informative
μ_{G_W}	N$(0.42, (1.18)^2)$	Weakly Informative
μ_P	N$(1.41, (1.81)^2)$	Weakly Informative
ρ_{G_W}	Uniform$(-1, 1)$	Uninformative
ρ_P	Uniform$(-1, 1)$	Uninformative
σ^2_η	Inv-gamma$(0.001, 0.001)$	Uninformative
$\sigma^2_{\eta C}$	Inv-gamma$(0.001, 0.001)$	Uninformative
$\sigma^2_{\eta D}$	Inv-gamma$(0.001, 0.001)$	Uninformative
$\sigma^2_{\eta B}$	Inv-gamma$(0.001, 0.001)$	Uninformative
$\sigma^2_{\eta R}$	Inv-gamma$(0.01, 0.01)$	Uninformative
$\sigma^2_{\eta H}$	Inv-gamma$(0.001, 0.001)$	Uninformative
$\sigma^2_{G_W}$	Inv-gamma$(0.001, 0.001)$	Uninformative
σ^2_W	Inv-gamma$(0.001, 0.001)$	Uninformative
σ^2_P	Inv-gamma$(0.001, 0.001)$	Uninformative
π_{DH}	Uniform$(0, 1)$	Uninformative
π_{RH}	Uniform$(0, 1)$	Uninformative
π_{HH}	Uniform$(0, 1)$	Uninformative
π_{BH}	Uniform$(0, 1)$	Uninformative
π_{DB}	Uniform$(0, 1)$	Uninformative
π_{RB}	Uniform$(0, 1)$	Uninformative
π_{HB}	Uniform$(0, 1)$	Uninformative
π_{BB}	Uniform$(0, 1)$	Uninformative
π_{CB}	Uniform$(0, 1)$	Uninformative
π_{BC}	Uniform$(0, 1)$	Uninformative
$\sigma^2_{\epsilon TOC}$	0.025	Fixed
$\sigma^2_{\epsilon POC}$	0.9	Fixed
$\sigma^2_{\epsilon G_W}$	0.023	Fixed
$\sigma^2_{\epsilon W}$	0.133	Fixed
$\sigma^2_{\epsilon P}$	0.067	Fixed
$\sigma^2_{\epsilon IOM}$	0.01	Fixed
$\sigma^2_{\epsilon H}$	0.1	Fixed

Table S2: Prior distributions of parameters of the Tarlee dataset and the ones are common in both datasets.

Parameter	Prior	Type
$X^1_{C(1981)}$	Truncated-normal$(60, 15^2, lower = 0)$	Uninformative
$X^2_{C(1981)}$	Truncated-normal$(60, 15^2, lower = 0)$	Uninformative
$X^3_{C(1981)}$	Truncated-normal$(60, 15^2, lower = 0)$	Uninformative
X_{IOM}	Truncated-normal$(12, 2^2, lower = 0)$	Uninformative
h_S	LN$(0.46, (1.6)^2)$	Informative
ρ_{G_S}	Uniform$(-1, 1)$	Uninformative
μ_{G_S}	N$(0.42, (1.18)^2)$	Weakly Informative
r_S	N$(0.5, (0.067)^2)$	Informative
K_B	Truncated-normal$(0.66, 0.3^2, lower = 0)$	Informative
K_R	Truncated-normal$(0.15, 0.075^2, lower = 0)$	Informative
$\sigma^2_{G_S}$	Inv-gamma$(0.001, 0.001)$	Uninformative
$\sigma^2_{\eta B}$	Truncated-normal$(0, 0.5^2, lower = 0)$	Weakly Informative
$\sigma^2_{\eta R}$	Truncated-normal$(0, 0.5^2, lower = 0)$	Weakly Informative
$\sigma^2_{\eta H}$	Truncated-normal$(0, 0.5^2, lower = 0)$	Weakly Informative
σ^2_S	Inv-gamma$(0.001, 0.001)$	Uninformative
$\sigma^2_{\epsilon G_S}$	0.023	Fixed
$\sigma^2_{\epsilon S}$	0.133	Fixed

Table S3: Prior distributions of parameters of the Brigalow dataset.

The proposal density functions of the one, two, and three-pool models applied on the Tarlee dataset are presented in Table S4. In the three-pool model, the proposal density functions of some parameters are different from the ones in the one and two-pool models, therefore, we show them in Table S5. The proposal density functions of the five-pool model related to the Tarlee dataset are shown in Table S6. Tables S7, S8, S9, and S10 show respectively the proposal density functions of the one, two, three, and five-pool models related to the Brigalow dataset. To avoid repetition, the proposal density functions of parameters which are the same in two or three-pool model are not shown in Tables S9 and S10.

C State-space Model

The state-space model uses observable measurement variable $Y_{(t)}$ and unobserved state variable $X_{(t)}$ which can be estimated through observational data that depend on the state variable, to describe a system. This model includes the first two levels of the hierarchy of

Parameter	Proposal
K_C	$\mathrm{N}(K_C, 0.001^2)$
K_B	Truncated-normal$(K_B, 0.09^2, lower = 0)$
c	Truncated-normal$(c, 0.005^2, lower = 0, upper = 1)$
r_W	Truncated-normal$(r_W, 0.05^2, lower = 0)$
r_P	Truncated-normal$(r_P, 0.05^2, lower = 0)$
p	Truncated-normal$(p, 0.005^2, lower = 0, upper = 1)$
h_W	Truncated-normal$(h_W, 0.05^2, lower = 0)$
μ_{G_W}	$\mathrm{N}(\mu_{G_W}, 0.05^2)$
μ_P	$\mathrm{N}(\mu_P, 0.05^2)$
ρ_{G_W}	Truncated-normal$(\rho_{G_W}, 0.05^2, lower = -1, upper = 1)$
ρ_P	Truncated-normal$(\rho_P, 0.1^2, lower = -1, upper = 1)$
σ^2_η	Truncated-normal$(\sigma^2_\eta, 0.001^2, lower = 0)$
$\sigma^2_{\eta C}$	Truncated-normal$(\sigma^2_{\eta C}, 0.001^2, lower = 0)$
$\sigma^2_{\eta B}$	Truncated-normal$(\sigma^2_{\eta B}, 0.01^2, lower = 0)$
$\sigma^2_{G_W}$	Truncated-normal$(\sigma^2_{G_W}, \frac{\sigma^2_{G_W}}{20^2}, lower = 0)$
σ^2_W	Truncated-normal$(\sigma^2_W, 0.001^2, lower = 0)$
σ^2_P	Truncated-normal$(\sigma^2_P, 0.1^2, lower = 0)$
π_{CB}	Truncated-normal$(\pi_{CB}, 0.05^2, lower = 0, upper = 1)$
π_{BC}	Truncated-normal$(\pi_{BC}, 0.1^2, lower = 0, upper = 1)$
π_{BB}	Truncated-normal$(\pi_{BB}, 0.1^2, lower = 0, upper = 1)$
$X^1_{C(1978)}$	Truncated-normal$(X^1_{C(1978)}, 5^2, lower = 0)$
$X^2_{C(1978)}$	Truncated-normal$(X^2_{C(1978)}, 5^2, lower = 0)$
$X^3_{C(1978)}$	Truncated-normal$(X^3_{C(1978)}, 5^2, lower = 0)$
X_{IOM}	Truncated-normal$(X_{IOM}, 0.9^2, lower = 0)$

Table S4: Proposal distributions of one, two, and three-pool models used in the CPM method for the Tarlee dataset.

Parameter	Proposal
K_C	$\mathrm{N}(K_C, 0.005^2)$
μ_G	$\mathrm{N}(\mu_G, 0.075^2)$
μ_P	$\mathrm{N}(\mu_P, 0.1^2)$
$X^1_{C(1978)}$	Truncated-normal$(X^1_{C(1978)}, 2^2, lower = 0)$
$X^2_{C(1978)}$	Truncated-normal$(X^2_{C(1978)}, 2^2, lower = 0)$
$X^3_{C(1978)}$	Truncated-normal$(X^3_{C(1978)}, 2^2, lower = 0)$
X_{IOM}	Truncated-normal$(X_{IOM}, 0.09^2, lower = 0)$

Table S5: Proposal distributions of some parameters in the three-pool model used in the CPM method for the Tarlee dataset.

the BHM framework and its generic representation with Gaussian noise is

$$\begin{aligned} X_{(t)} &= f(X_{(t-1)}) + \boldsymbol{B}u_{(t)} + \epsilon_{(t)} \\ Y_{(t)} &= g(X_{(t)}) + \nu_{(t)}; \end{aligned} \tag{1}$$

Parameter	Proposal
K_D	Truncated-normal$(K_D, 0.005^2, lower = 0)$
K_B	Truncated-normal$(K_B, 0.09^2, lower = 0)$
K_R	Truncated-normal$(K_R, 0.005^2, lower = 0)$
K_H	Truncated-normal$(K_H, 0.006^2, lower = 0)$
c	Truncated-normal$(c, 0.005^2, lower = 0, upper = 1)$
r_W	Truncated-normal$(r_W, 0.05^2, lower = 0)$
r_P	Truncated-normal$(r_P, 0.05^2, lower = 0)$
p	Truncated-normal$(p, 0.005^2, lower = 0, upper = 1)$
h	Truncated-normal$(h, 0.05^2, lower = 0)$
μ_G	N$(K, 0.075^2)$
μ_P	N$(K, 0.1^2)$
ρ_G	Truncated-normal$(\rho_G, 0.25^2, lower = -1, upper = 1)$
ρ_P	Truncated-normal$(\rho_P, 0.2^2, lower = -1, upper = 1)$
$\sigma^2_{\eta D}$	Truncated-normal$(\sigma^2_{\eta D}, 0.1^2, lower = 0)$
σ^2_G	Truncated-normal$(\sigma^2_G, \frac{\sigma^2_G}{20^2}, lower = 0)$
σ^2_W	Truncated-normal$(\sigma^2_W, 0.01^2, lower = 0)$
σ^2_P	Truncated-normal$(\sigma^2_P, 0.1^2, lower = 0)$
X_{IOM}	Truncated-normal$(X_{IOM}, 0.09^2, lower = 0)$
$X^1_{C(1978)}$	Truncated-normal$(X^1_{C(1978)}, 1.3^2, lower = 0)$
$X^2_{C(1978)}$	Truncated-normal$(X^2_{C(1978)}, 1.3^2, lower = 0)$
$X^3_{C(1978)}$	Truncated-normal$(X^3_{C(1978)}, 1.3^2, lower = 0)$
$\sigma^2_{\eta B}$	Truncated-normal$(\sigma^2_B, 0.1^2, lower = 0)$
P_D	Truncated-normal$(P_D, 0.15^2, lower = 0, upper = 1)$
σ^2_R	Truncated-normal$(\sigma^2_R, 0.09^2, lower = 0)$
σ^2_H	Truncated-normal$(\sigma^2_H, 0.01^2, lower = 0)$
π_{DB}	Truncated-normal$(\pi_{DB}, 0.05^2, lower = 0, upper = 1)$
π_{BB}	Truncated-normal$(\pi_{BB}, 0.15^2, lower = 0, upper = 1)$
π_{DH}	Truncated-normal$(\pi_{DH}, 0.09^2, lower = 0, upper = 1)$
π_{RH}	Truncated-normal$(\pi_{RH}, 0.1^2, lower = 0, upper = 1)$
π_{HH}	Truncated-normal$(\pi_{HH}, 0.1^2, lower = 0, upper = 1)$
π_{BH}	Truncated-normal$(\pi_{BH}, 0.015^2, lower = 0, upper = 1)$
π_{RB}	Truncated-normal$(\pi_{RB}, 0.09^2, lower = 0, upper = 1)$
π_{HB}	Truncated-normal$(\pi_{HB}, 0.1^2, lower = 0, upper = 1)$

Table S6: Proposal distributions of the five-pool model used in the CPM method for the Tarlee dataset.

where $\epsilon_{(t)} \sim N(\mu, \sigma^2_X)$ and $\nu_{(t)} \sim N(\lambda, \sigma^2_Y)$ are state and measurement noise components, respectively and the control-input matrix $\boldsymbol{B}$ is applied to a known vector of inputs $u_{(t)}$. The aim is to produce estimators for the state variable $X_{(t)}$ through the filtering distribution $p(X_{(t)}|Y_{1:t})$. When the filtering distribution does not have a closed-form expression some methods such as the Kalman filter (KF) (in the case of linear-Gaussian model) and particle filter can be used to approximate it. For the sake of simplicity, we assume the static parameter $\boldsymbol{\theta}$ is fixed in this section.

Parameter	Proposal
K_C	$\text{N}(K_C, 0.002^2)$
c	Truncated-normal$(c, 0.005^2, lower = 0, upper = 1)$
r_W	Truncated-normal$(r_W, 0.05^2, lower = 0)$
p	Truncated-normal$(p, 0.005^2, lower = 0, upper = 1)$
h_W	Truncated-normal$(h_W, 0.05^2, lower = 0)$
μ_{G_W}	$\text{N}(\mu_{G_W}, 0.05^2)$
ρ_{G_W}	Truncated-normal$(\rho_{G_W}, 0.05^2, lower = -1, upper = 1)$
σ_η^2	Truncated-normal$(\sigma_\eta^2, 0.001^2, lower = 0)$
$\sigma_{G_W}^2$	Truncated-normal$(\sigma_{G_W}^2, \frac{\sigma_{G_W}^2}{20^2}, lower = 0)$
σ_W^2	Truncated-normal$(\sigma_W^2, 0.001^2, lower = 0)$
$X_{C(1981)}^1$	Truncated-normal$(X_{C(1981)}^1, 2^2, lower = 0)$
$X_{C(1981)}^2$	Truncated-normal$(X_{C(1981)}^2, 2^2, lower = 0)$
$X_{C(1981)}^3$	Truncated-normal$(X_{C(1981)}^3, 2^2, lower = 0)$
r_S	Truncated-normal$(r_S, 0.05^2, lower = 0)$
σ_S^2	Truncated-normal$(\sigma_S^2, 0.05^2, lower = 0)$
μ_{G_S}	$\text{N}(\mu_{G_S}, 0.05^2)$
ρ_{G_S}	Truncated-normal$(\rho_{G_S}, 0.25^2, lower = -1, upper = 1)$
$\sigma_{G_S}^2$	Truncated-normal$(\sigma_{G_S}^2, \frac{\sigma_{G_S}^2}{20^2}, lower = 0)$
h_S	Truncated-normal$(h_S, 0.25^2, lower = 0)$

Table S7: Proposal distributions of one-pool models used in the CPM method for the Brigalow dataset.

Parameter	Proposal
K_C	$\text{N}(K_C, 0.01^2)$
c	Truncated-normal$(c, 0.005^2, lower = 0, upper = 1)$
r_W	Truncated-normal$(r_W, 0.05^2, lower = 0)$
p	Truncated-normal$(p, 0.005^2, lower = 0, upper = 1)$
h_W	Truncated-normal$(h_W, 0.1^2, lower = 0)$
μ_{G_W}	$\text{N}(\mu_{G_W}, 0.05^2)$
ρ_{G_W}	Truncated-normal$(\rho_{G_W}, 0.05^2, lower = -1, upper = 1)$
σ_η^2	Truncated-normal$(\sigma_\eta^2, 0.001^2, lower = 0)$
$\sigma_{G_W}^2$	Truncated-normal$(\sigma_{G_W}^2, \frac{\sigma_{G_W}^2}{20^2}, lower = 0)$
σ_W^2	Truncated-normal$(\sigma_W^2, 0.01^2, lower = 0)$
$X_{C(1981)}^1$	Truncated-normal$(X_{C(1981)}^1, 2^2, lower = 0)$
$X_{C(1981)}^2$	Truncated-normal$(X_{C(1981)}^2, 2^2, lower = 0)$
$X_{C(1981)}^3$	Truncated-normal$(X_{C(1981)}^3, 2^2, lower = 0)$
X_{IOM}	Truncated-normal$(X_{IOM}, 0.05^2, lower = 0)$
r_S	Truncated-normal$(r_S, 0.05^2, lower = 0)$
σ_S^2	Truncated-normal$(\sigma_S^2, 0.01^2, lower = 0)$
μ_{G_S}	$\text{N}(\mu_{G_S}, 0.05^2)$
ρ_{G_S}	Truncated-normal$(\rho_{G_S}, 0.25^2, lower = -1, upper = 1)$
$\sigma_{G_S}^2$	Truncated-normal$(\sigma_{G_S}^2, \frac{\sigma_{G_S}^2}{20^2}, lower = 0)$
h_S	Truncated-normal$(h_S, 0.25^2, lower = 0)$

Table S8: Proposal distributions of two-pool models used in the CPM method for the Brigalow dataset.

Parameter	Proposal
$\sigma^2_{\eta_C}$	Truncated-normal$(\sigma^2_{\eta_C}, 0.001^2, lower = 0)$
σ^2_B	Truncated-normal$(\sigma^2_B, 0.1^2, lower = 0)$
K_B	Truncated-normal$(K_B, 0.09^2, lower = 0)$
π_{DB}	Truncated-normal$(\pi_{DB}, 0.1^2, lower = 0)$
π_{BB}	Truncated-normal$(\pi_{BB}, 0.1^2, lower = 0)$
π_{BC}	Truncated-normal$(\pi_{BC}, 0.1^2, lower = 0)$
π_{CB}	Truncated-normal$(\pi_{CB}, 0.05^2, lower = 0)$
K_R	Truncated-normal$(K_R, 0.1^2, lower = 0)$

Table S9: Proposal distributions of three-pool models used in the CPM method for the Brigalow dataset.

Parameter	Proposal
$\sigma^2_{\eta_D}$	Truncated-normal$(\sigma^2_{\eta_D}, 0.02^2, lower = 0)$
$\sigma^2_{\eta_B}$	Truncated-normal$(\sigma^2_{\eta_B}, 0.1^2, lower = 0)$
$\sigma^2_{\eta_R}$	Truncated-normal$(\sigma^2_{\eta_R}, 0.9^2, lower = 0)$
$\sigma^2_{\eta_H}$	Truncated-normal$(\sigma^2_{\eta_H}, 0.1^2, lower = 0)$
$\sigma^2_{G_W}$	Truncated-normal$(\sigma^2_{G_W}, \frac{\sigma^2_{G_W}}{30^2}, lower = 0)$
$\sigma^2_{G_S}$	Truncated-normal$(\sigma^2_{G_S}, \frac{\sigma^2_{G_W}}{10^2}, lower = 0)$
σ^2_S	Truncated-normal$(\sigma^2_S, 0.05^2, lower = 0)$
X_{IOM}	Truncated-normal$(X_{IOM}, 0.9^2, lower = 0)$
$X^1_{C(1981)}$	Truncated-normal$(X^1_{C(1981)}, 3^2, lower = 0)$
$X^2_{C(1981)}$	Truncated-normal$(X^2_{C(1981)}, 3^2, lower = 0)$
$X^3_{C(1981)}$	Truncated-normal$(X^3_{C(1981)}, 3^2, lower = 0)$
K_D	Truncated-normal$(K_D, 0.005^2, lower = 0)$
K_B	Truncated-normal$(K_B, 0.1^2, lower = 0)$
K_R	Truncated-normal$(K_R, 0.05^2, lower = 0)$
K_H	Truncated-normal$(K_H, 0.01^2, lower = 0)$
h_W	Truncated-normal$(h_W, 0.05^2, lower = 0)$
h_S	Truncated-normal$(h_S, 0.5^2, lower = 0)$
P_D	Truncated-normal$(P_D, 0.15^2, lower = 0)$
π_{DH}	Truncated-normal$(\pi_{DH}, 0.09^2, lower = 0)$
π_{RH}	Truncated-normal$(\pi_{RH}, 0.1^2, lower = 0)$
π_{HH}	Truncated-normal$(\pi_{HH}, 0.1^2, lower = 0)$
π_{BH}	Truncated-normal$(\pi_{BH}, 0.15^2, lower = 0)$
π_{RB}	Truncated-normal$(\pi_{RB}, 0.09^2, lower = 0)$
π_{HB}	Truncated-normal$(\pi_{HB}, 0.1^2, lower = 0)$
ρ_{G_S}	Truncated-normal$(\rho_{G_S}, 0.1^2, lower = -1, upper = 1)$

Table S10: Proposal distributions of five-pool models used in the CPM method for the Brigalow dataset.

C.1 Kalman Filter

In the case of linear-Gaussian state-space model, the KF can be used to estimate state variable as an efficient method since it is an optimal estimator in the sense of minimising

the variance of the estimated state. The linear-Gaussian state-space model has the form

$$X_{(t)} = \boldsymbol{A}^* X_{(t-1)} + \boldsymbol{B}^* u_{(t)} + \epsilon^*_{(t)}$$
$$Y_{(t)} = \boldsymbol{C}^* X_{(t)} + \nu^*_{(t)};$$

where $\epsilon^*_{(t)} \sim N(\mathbf{0}, \boldsymbol{Q}^*)$, $\nu^*_{(t)} \sim N(\mathbf{0}, \boldsymbol{R}^*)$, $\boldsymbol{A}^*$ is the state-transition matrix, the control-input matrix $\boldsymbol{B}^*$ is applied to a known vector of inputs $u_{(t)}$, and $\boldsymbol{C}^*$ is the observation matrix. The KF method is shown in Algorithm S1, here MVN$(\boldsymbol{\mu}, \boldsymbol{\Sigma})$ denotes the multivariate normal density with mean vector $\boldsymbol{\mu}$ and covariance matrix $\boldsymbol{\Sigma}$. In Algorithm S1, $\boldsymbol{K}_{(t)}$ is the Kalman gain matrix, $\boldsymbol{P}^{t-1}_{(t)}$ and $X^{t-1}_{(t)}$ are the process noise and the expectations of state variable, respectively given all observations up to and including time $t-1$.

Algorithm S1 Kalman filter algorithm

1: Initialize with initial state $\hat{X}_{(0)} = x_{(0)}$ and $\hat{\boldsymbol{P}}_{(0)} = \boldsymbol{Q}^*$ at $t = 0$;
2: **for** $t = 1, ..., T$ **do**
3: $X^{t-1}_{(t)} = \boldsymbol{A}^* \hat{X}_{(t-1)} + \boldsymbol{B}^* u_{(t)}$, State estimate extrapolation;
4: $\boldsymbol{P}^{t-1}_{(t)} = \boldsymbol{A}^* \hat{\boldsymbol{P}}_{(t-1)} \boldsymbol{A}^{*'} + \boldsymbol{Q}^*$, State covariance extrapolation;
5: $\boldsymbol{K}_{(t)} = \boldsymbol{P}^{t-1}_{(t)} \boldsymbol{C}^{*'} [\boldsymbol{R}^* + \boldsymbol{C}^* \boldsymbol{P}^{t-1}_{(t)} \boldsymbol{C}^{*'}]^{-1}$, Kalman gain matrix;
6: $\hat{X}_{(t)} = X^{t-1}_{(t)} + \boldsymbol{K}_{(t)} [Y_{(t)} - \boldsymbol{C}^* X^{t-1}_{(t)}]$, State estimate update;
7: $\hat{\boldsymbol{P}}_{(t)} = [\boldsymbol{I} - \boldsymbol{K}_{(t)} \boldsymbol{C}^*] \boldsymbol{P}^{t-1}_{(t)}$, State covariance update;
8: Compute the log-likelihood contribution, $l^{\text{KF}}_{(t)}$, at time t through the density MVN$(Y_{(t)} - \boldsymbol{C}^* X^{t-1}_{(t)}, \boldsymbol{R}^* + \boldsymbol{C}^* \boldsymbol{P}^{t-1}_{(t)} \boldsymbol{C}^{*'})$;
9: The complete log-likelihood can be calculated as $L^* = \sum_t l^{\text{KF}}_{(t)}$

To apply the KF to the Tarlee model, we use a log transformation, i.e. $X^* = \log(X)$ and $Y^* = \log(Y)$ in order to form a linear-Gaussian state-space model for the state-space model formed by the sub-model involving $\{X^*_G, X^*_W, X^*_P, Y^*_G, Y^*_W, Y^*_P\}$. Since $X^*_{W(t)}$ depends on $X^*_{G(t)}$ and because of the auto-regressive structure of the state-space model, we rewrite $X^*_{W(t)}$ as follows

$$X^{*i}_{W(t)} \sim N(\log h + \mu_G + \rho_G(X^{*i}_{G(t-1)} - \mu_G), \sigma^2_W + \sigma^2_G).$$

We can produce estimators of the state variables in the case of linear and non-linear state-space models through particle filters. The algorithm of one of particle filters is introduced

in the next section.

C.2 Bootstrap Particle Filter

In the linear and non-linear cases, particle filters can be used to produce estimators of the state variable $X_{(t)}$ and the simplest form of particle filters known as bootstrap particle filter is applied on non-linear part of the model in this study. The algorithm of bootstrap particle filter is provided in Algorithm S2.

Algorithm S2 Bootstrap particle filter algorithm

for $k = 1, ..., N$ **do**
  $t = 1$, draw sample $X^k_{(1)} \sim p(X_{(1)})$;
for $t = 2, ..., T$ **do**
  for $k = 1, ..., N$ **do**
    Draw sample $X^k_{(t)} \sim p(X_{(t)}|X^{*k}_{(t-1)})$;
    Calculate weights $w^k_{(t)} = p(Y_{(t)}|X^k_{(t)})$;
  Estimate the log-likelihood component for the tth observation, $\hat{l}_{(t)} = \log \left(\frac{\sum_j w^j_{(t)}}{N} \right)$;
  Normalise weights $W^k_{(t)} = \frac{w^k_{(t)}}{\sum_j w^j_{(t)}}$ for $k \in \{1, 2, \ldots, N\}$;
  Resample with replacement N particles $X^k_{(t)}$ based on the normalised importance weights;
  Estimate the overall log-likelihood $L^* = \sum_t \hat{l}_{(t)}$.

C.3 Correlated Pseudo-marginal Method

When the process and observation models depend on a set of unknown static parameters $\boldsymbol{\theta}$, one can treat the parameters as random variables and utilise Bayesian approach to estimate $\boldsymbol{\theta}$. In the case of having an intractable posterior distribution, numerical methods such as Markov chain Monte Carlo (MCMC) methods can be used. In this study, we utilise correlated pseudo-marginal method, one of the MCMC methods, to generate a sequence of correlated random samples from a probability distribution from which direct sampling is difficult. The likelihood estimators $\hat{p}(\mathbf{Y}|\mathbf{X}, \boldsymbol{\theta}^*)$ and $\hat{p}(\mathbf{Y}|\mathbf{X}, \boldsymbol{\theta}_{m-1})$ in the acceptance ratio of the CPM method are correlated through correlating the auxiliary random numbers U, used to obtain these estimators, in order to reduce the variance of the resulting ratio. The CPM algorithm is presented in Algorithm S3. To have a highly correlated likelihood estimators in

the CPM algorithm it is required to use a particle filter that processes the random numbers in such a manner that the likelihood estimates are similar as possible when slightly perturbing the random numbers. Algorithm S4 shows the particle filter with a given set of random numbers.

Algorithm S3 Correlated pseudo-marginal algorithm

Initialise $\boldsymbol{\theta}_0$;
for $m = 1, ..., M^*$ **do**
  Sample $\boldsymbol{\theta}^* \sim Q(.|\boldsymbol{\theta}_{m-1})$;
  Sample $\xi \sim N(\mathbf{0}, \boldsymbol{I})$ and set $U^* = \tau U_{m-1} + \sqrt{1-\tau^2}\xi$;
  Compute the estimator $\hat{p}(\mathbf{Y}|\boldsymbol{\theta}^*, U^*)$ using Algorithm S4
  Compute the acceptance ratio:

$$r = \frac{\hat{p}(\mathbf{Y}|\boldsymbol{\theta}^*, U^*)p(\boldsymbol{\theta}^*)Q(\boldsymbol{\theta}_{m-1}|\boldsymbol{\theta}^*)}{\hat{p}(\mathbf{Y}|\boldsymbol{\theta}_{m-1}, U_{m-1})p(\boldsymbol{\theta}_{m-1})Q(\boldsymbol{\theta}^*|\Theta_{m-1})};$$

  Accept $(\boldsymbol{\theta}^*, U^*)$ with probability $\min(r, 1)$ otherwise, output $(\boldsymbol{\theta}_{m-1}, U_{m-1})$

Algorithm S4 Particle filter with fixed random numbers

Sample $U_{(j^*)} \sim N(0,1)$ and $V_{(i^*)} \sim N(0,1)$ for all $j^* \in \{1, \ldots, TN\}$ and $i^* \in \{1, \ldots, T\}$;
Sample $X^k_{(1)} \sim p(.|U_{1:N}, \boldsymbol{\theta})$ for all $k \in \{1, \ldots, N\}$;
for $t = 1, ..., T\text{-}1$ **do**
  Sort the collection $\{X^1_{(t)}, \ldots, X^N_{(t)}\}$;
  Compute importance weights $w^k_{(t)}$ and log-likelihoods $\hat{l}_{(t)} = \log\left(\frac{\sum_k w^k_{(t)}}{N}\right)$ for $k \in \{1, \ldots, N\}$;
  Sample $X^k_{(t)}$ based on systematic resampling using random values $V_{1:T}$ and normalised weights $W^k_{(t)}$ for $k \in \{1, \ldots, N\}$;
  Set $X^k_{(t+1)}$ as a sample from $p(.|X^k_{(t)}, U_{Nt+1:N(t+1)}, \boldsymbol{\theta})$ for $k \in \{1, \ldots, N\}$;
Estimate the overall log-likelihood $L^* = \sum_t \hat{l}_{(t)}$.

The process and observation models of the Tarlee and Brigalow datasets are presented in the next sections.

D One-pool Model

D.1 Process Model

The process model of the one-pool model at time t in field (or soil type) i is:

$$\log(X^i_{C(t)}) = \log(X^i_{C(t-1)}e^{-K_C\Delta t} + I^i_{C(t)}) + \eta^i_{(t)}, \quad \eta^i_{(t)} \sim N(0, \sigma^2_\eta); \tag{2}$$

$$X^i_{G_W(t)} \sim LN(\mu_{G_W} + \rho_{G_W}(\log(X^i_{G_W(t-1)}) - \mu_{G_W}), \sigma^2_{G_W}); \tag{3}$$

$$X^i_{W(t)} \sim LN(\log h_W + \log(x^i_{G_W(t)}), \sigma^2_W); \tag{4}$$

$$X^i_{P(t)} \sim LN(\mu_P + \rho_P(\log(X^i_{P(t-1)}) - \mu_P), \sigma^2_P); \tag{5}$$

$$X^i_{G_S(t)} \sim LN(\mu_{G_S} + \rho_{G_S}(\log(X^i_{G_S(t-1)}) - \mu_{G_S}), \sigma^2_{G_S}); \quad \text{and} \tag{6}$$

$$X^i_{S(t)} \sim LN(\log h_S + \log(x^i_{G_S(t)}), \sigma^2_S); \tag{7}$$

where h_W, h_S, ρ_P, ρ_{G_W}, and ρ_{G_S} denote the harvest index which is the ratio of wheat to grain, the harvest index which is the ratio of sorghum to grain, auto-regressive parameters for the evolution of pasture total dry matter (TDM) and grain TDM of wheat and sorghum, respectively. Notice that $X^i_{W(t)}$ and $X^i_{S(t)}$ are defined conditional on $X^i_{G_W(t)} = x^i_{G_W(t)}$ and $X^i_{G_S(t)} = x^i_{G_S(t)}$, respectively. The process of total wheat and sorghum dry matters and the total grain dry matters are separated because of measuring grain yield in spite of that the total wheat and sorghum dry matter contain the total grain dry matters (i.e. $X^i_{G_W(t)} \leq X^i_{W(t)}$ and $X^i_{G_S(t)} \leq X^i_{S(t)}$). Notice that since there is no sorghum crop in the Tarlee dataset, we do not consider process and measurement models of the sorghum in its model. In addition, as there is no pasture in the Brigalow dataset, we do not consider process and measurement models of the pasture in the model. Therefore, the mass of carbon inputs, $I^i_{C(t)}$, of the Tarlee

and Brigalow datasets which are denoted by $IT^i_{C(t)}$ and $IB^i_{C(t)}$ respectively are:

$$IT^i_{C(t)} = \begin{cases} c(X^i_{W(t)} - X^i_{G_W(t)}) + cr_W X^i_{W(t)} & \text{Wheat for Grain} \\ cpX^i_{W(t)} + cr_W X^i_{W(t)} & \text{Wheat for Hay} \\ cX^i_{P(t)} + cr_P X^i_{P(t)} & \text{Pasture} \\ cpX^i_{P(t)} + cr_P X^i_{P(t)} & \text{Pasture for Hay} \\ 0 & \text{Fallow} \end{cases}$$

and

$$IB^i_{C(t)} = \begin{cases} c(X^i_{W(t)} - X^i_{G_W(t)}) + cr_W X^i_{W(t)} & \text{Wheat for Grain} \\ cpX^i_{W(t)} + cr_W X^i_{W(t)} & \text{Wheat for Hay} \\ c(X^i_{S(t)} - X^i_{G_S(t)}) + cr_S X^i_{S(t)} & \text{Sorghum for Grain} \\ cpX^i_{S(t)} + cr_S X^i_{S(t)} & \text{Sorghum for Hay} \\ 0 & \text{Fallow} \end{cases}$$

where p, r_W, r_S, and r_P are the proportion of the crop left above-ground after harvest, the root-to-shoot ratios (in terms of TDM) for wheat, sorghum and pasture crops, respectively. The amount of carbon that enters into the soil from plant-matter that is already below-ground (i.e., roots) and from plant-matter that remains above-ground after harvesting, $c(X^i_{W(t)} - X^i_{G_W(t)})$ and $c(X^i_{S(t)} - X^i_{G_S(t)})$, are included in $IT^i_{C(t)}$ and $IB^i_{C(t)}$, respectively. Here c, $(X^i_{S(t)} - X^i_{G_S(t)})$ and $(X^i_{W(t)} - X^i_{G_W(t)})$ are the carbon content of dry plant matter and the above-ground plant-matter biomass of sorghum and wheat, respectively.

In this model, $X^i_{(t)} = (X^i_{C(t)}, X^i_{G_W(t)}, X^i_{W(t)}, X^i_{P(t)}, X^i_{G_S(t)}, X^i_{S(t)})$ is all processes at time t in field (soil type) i. Given a vector of parameters $\boldsymbol{\theta}$ for the model, the transition density of the joint process model (which is a discrete-time Markov chain) of the independent processes

(2)-(7) can be written as:

$$p(X^i_{(t)}|X^i_{(t-1)}, \boldsymbol{\theta}) = p(X^i_{C(t)}|I^i_{C(t)}, X^i_{C(t-1)}, \boldsymbol{\theta}) \times p(X^i_{G_W(t)}|X^i_{G_W(t-1)}, \boldsymbol{\theta})$$
$$\times\, p(X^i_{W(t)}|X^i_{G_W(t)}, \boldsymbol{\theta}) \times p(X^i_{P(t)}|X^i_{P(t-1)}, \boldsymbol{\theta}) \times p(X^i_{G_S(t)}|X^i_{G_S(t-1)}, \boldsymbol{\theta})$$
$$\times\, p(X^i_{S(t)}|X^i_{G_S(t)}, \boldsymbol{\theta}).$$

The overall transition density is

$$p(X_{(t)}|X_{(t-1)}, \boldsymbol{\theta}) = \prod_{i=1}^{3} p(X^i_{(t)}|X^i_{(t-1)}, \boldsymbol{\theta});$$

as the three fields (soil types) are independent. For the sake of simplicity, we use the same notation for the overall transition density in all models.

D.2 Observation Model

The observation model of the one-pool includes the sub-models (8)-(13) to account for measurement error.

$$Y^i_{TOC(t)}|X^i_{C(t)} = x^i_{C(t)} \sim LN(\log(x^i_{C(t)}), \sigma^2_{\epsilon TOC}); \tag{8}$$
$$Y^i_{G_W(t)}|X^i_{G_W(t)} = x^i_{G_W(t)} \sim LN(\log(x^i_{G_W(t)}), \sigma^2_{\epsilon G_W}); \tag{9}$$
$$Y^i_{W(t)}|X^i_{W(t)} = x^i_{W(t)} \sim LN(\log(x^i_{W(t)}), \sigma^2_{\epsilon W}); \tag{10}$$
$$Y^i_{P(t)}|X^i_{P(t)} = x^i_{P(t)} \sim LN(\log(x^i_{P(t)}), \sigma^2_{\epsilon P}); \tag{11}$$
$$Y^i_{G_S(t)}|X^i_{G_S(t)} = x^i_{G_S(t)} \sim LN(\log(x^i_{G_S(t)}), \sigma^2_{\epsilon G_S}); \quad \text{and} \tag{12}$$
$$Y^i_{S(t)}|X^i_{S(t)} = x^i_{S(t)} \sim LN(\log(x^i_{S(t)}), \sigma^2_{\epsilon S}). \tag{13}$$

As the measurements (8)-(13) are independent, the joint observation model of them at time t and field (or soil type) i, conditioning on unobserved variable vector $X^i_{(t)}$ and parameter

$\boldsymbol{\theta}$ is:

$$
\begin{aligned}
p(Y^i_{(t)}|X^i_{(t)},\boldsymbol{\theta}) = p(Y^i_{TOC(t)}|X^i_{C(t)},\boldsymbol{\theta}) \times p(Y^i_{G_W(t)}|X^i_{G_W(t)},\boldsymbol{\theta}) \\
\times p(Y^i_{W(t)}|X^i_{W(t)},\boldsymbol{\theta}) \times p(Y^i_{P(t)}|X^i_{P(t)},\boldsymbol{\theta}) \\
\times p(Y^i_{G_S(t)}|X^i_{G_S(t)},\boldsymbol{\theta}) \times p(Y^i_{S(t)}|X^i_{S(t)},\boldsymbol{\theta}); \qquad (14)
\end{aligned}
$$

where $Y^i_{(t)} = (Y^i_{TOC(t)}, Y^i_{G_W(t)}, Y^i_{W(t)}, Y^i_{P(t)}, Y^i_{G_S(t)}, Y^i_{S(t)})$. The overall observation model across all i's is therefore:

$$p(Y_{(t)}|X_{(t)},\boldsymbol{\theta}) = \prod_{i=1}^{3} p(Y^i_{(t)}|X^i_{(t)},\boldsymbol{\theta}).$$

For notational convenience, we use the same notation $Y_{(t)}$ and $X_{(t)}$ in other models presented below.

E Two-pool Model

E.1 Process Model

The process model of the two-pool model includes the following sub-models

$$
\begin{aligned}
&\log(X^i_{C(t)}) = \log(X^i_{C(t-1)}e^{-K_C\Delta t} + I^i_{C(t)}) + \eta^i_{(t)}, \quad \eta^i_{(t)} \sim N(0,\sigma^2_\eta); \\
&X^i_{G_W(t)} \sim LN(\mu_{G_W} + \rho_{G_W}(\log(X^i_{G_W(t-1)}) - \mu_{G_W}), \sigma^2_{G_W}); \\
&X^i_{W(t)} \sim LN(\log h_W + \log(x^i_{G_W(t)}), \sigma^2_W); \\
&X^i_{P(t)} \sim LN(\mu_P + \rho_P(\log(X^i_{P(t-1)}) - \mu_P), \sigma^2_P); \\
&X^i_{G_S(t)} \sim LN(\mu_{G_S} + \rho_{G_S}(\log(X^i_{G_S(t-1)}) - \mu_{G_S}), \sigma^2_{G_S}); \\
&X^i_{S(t)} \sim LN(\log h_S + \log(x^i_{G_S(t)}), \sigma^2_S); \quad \text{and} \\
&X^i_{IOM(t)} = X^i_{IOM(t-1)} = M; \qquad (15)
\end{aligned}
$$

where M is an unknown constant value.

E.2 Observation Model

The observation model of the two-pool model includes the following sub-models

$$Y^i_{TOC(t)}|X^i_{C(t)} = x^i_{C(t)}, X^i_{IOM(t)} = x^i_{IOM(t)} \sim LN(\log(x^i_{C(t)} + x^i_{IOM(t)}), \sigma^2_{\epsilon TOC}); \tag{16}$$

$$Y^i_{IOM(t)}|X^i_{IOM(t)} = x^i_{IOM(t)} \sim LN(\log(x^i_{IOM(t)}), \sigma^2_{\epsilon IOM}); \tag{17}$$

$$Y^i_{G_W(t)}|X^i_{G_W(t)} = x^i_{G_W(t)} \sim LN(\log(x^i_{G_W(t)}), \sigma^2_{\epsilon G_W});$$

$$Y^i_{W(t)}|X^i_{W(t)} = x^i_{W(t)} \sim LN(\log(x^i_{W(t)}), \sigma^2_{\epsilon W});$$

$$Y^i_{P(t)}|X^i_{P(t)} = x^i_{P(t)} \sim LN(\log(x^i_{P(t)}), \sigma^2_{\epsilon P});$$

$$Y^i_{G_S(t)}|X^i_{G_S(t)} = x^i_{G_S(t)} \sim LN(\log(x^i_{G_S(t)}), \sigma^2_{\epsilon G_S}); \quad \text{and}$$

$$Y^i_{S(t)}|X^i_{S(t)} = x^i_{S(t)} \sim LN(\log(x^i_{S(t)}), \sigma^2_{\epsilon S}).$$

As it is shown in (16), the observational data TOC depends on the state variables $X^i_{C(t)}$ and $X^i_{IOM(t)}$. Since the measurements (16) and (17) are independent, we compute the joint observation model of the components of this model by multiplying the probability density functions of measurement variables $Y^i_{TOC(t)}, Y^i_{G_W(t)}, Y^i_{W(t)}, Y^i_{P(t)}, Y^i_{G_S(t)}, Y^i_{S(t)}$, and $Y^i_{IOM(t)}$ given their corresponding state variables at time t.

F Three-pool Model

F.1 Process Model

The first pool of this model encompasses components DPM, POC, and HUM. To simplify notation, we keep the latent variable of this pool as $X^i_{C(t)}$ at time t in field (soil type) i. The process model of the three-pool model includes the latent variables $X^i_{B(t)}$ and $X^i_{C(t)}$ along with the latent variables of processes (15) and (3)-(7). The processes of $X^i_{C(t)}$ and $X^i_{B(t)}$ are

shown as

$$
\begin{aligned}
\log(X^i_{C(t)}) =& \log(X^i_{C(t-1)}e^{-K_C\Delta t} + I^i_{C(t)} \\
&+ X^i_{B(t-1)}(1-e^{-K_B\Delta t})\pi_{BC}) + \eta^i_{C(t)}, \quad \eta^i_{C(t)} \sim N(0,\sigma^2_{\eta_C}); \qquad (18) \\
\log(X^i_{B(t)}) =& \log(X^i_{B(t-1)}e^{-K_B\Delta t} + X^i_{C(t-1)}(1-e^{-K_C\Delta t})\pi_{CB} \\
&+ X^i_{B(t-1)}(1-e^{-K_B\Delta t})\pi_{BB}) + \eta^i_{B(t)}, \quad \eta^i_{B(t)} \sim N(0,\sigma^2_{\eta_B}). \qquad (19)
\end{aligned}
$$

Notice that the mass of BIO should be less than 5 percent of the total mass of carbon (e.g. $X_{BIO} \leq 0.05X_C$). The transition density of the joint process model of the latent variables in the three-pool model is the product of the transition densities of all state variables in the three-pool model.

F.2 Observation Model

The BIO pool is considered as the third pool in the three-pool model and uncertainty around the observations of the total SOC in field (soil type) i at time t is modeled as follows

$$
\begin{aligned}
Y^i_{TOC(t)}|X^i_{C(t)} = x^i_{C(t)}, X^i_{IOM(t)} = x^i_{IOM(t)} \\
, X^i_{B(t)} = x^i_{B(t)} \sim LN(\log(x^i_{C(t)} + x^i_{IOM(t)} + x^i_{B(t)}), \sigma^2_{\epsilon TOC}). \qquad (20)
\end{aligned}
$$

In addition to sub-model (20), the observation model of the three-pool model includes sub-models (9)-(13) and (17). The joint observation model of the three-pool model can be computed by multiplying the probability density functions of equations (9)-(13), (17), and (20). The next section introduces the process and observation models of the five-pool model in which each SOC component is considered as a pool.

G Five-pool Model

G.1 Process Model

The process model of the five-pool model includes sub-models (15), (3)-(7), and the following sub-models

$$\log(X^i_{D(t)}) = \log(X^i_{D(t-1)} e^{-K_D \Delta t} + P_D I^i_{C(t)}) + \eta^i_{D(t)}, \quad \eta^i_{D(t)} \sim N(0, \sigma^2_{\eta_D}); \tag{21}$$

$$\log(X^i_{R(t)}) = \log(X^i_{R(t-1)} e^{-K_R \Delta t} + (1 - P_D) I^i_{C(t)}) + \eta^i_{R(t)}, \quad \eta^i_{R(t)} \sim N(0, \sigma^2_{\eta_R}); \tag{22}$$

$$\begin{aligned} \log(X^i_{H(t)}) = &\log(X^i_{H(t-1)} e^{-K_H \Delta t} + X^i_{D(t-1)}(1 - e^{-K_D \Delta t})\pi_{DH} \\ &+ X^i_{R(t-1)}(1 - e^{-K_R \Delta t})\pi_{RH} + X^i_{H(t-1)}(1 - e^{-K_H \Delta t})\pi_{HH} \\ &+ X^i_{B(t-1)}(1 - e^{-K_B \Delta t})\pi_{BH}) + \eta^i_{H(t)}, \quad \eta^i_{H(t)} \sim N(0, \sigma^2_{\eta_H}); \end{aligned} \tag{23}$$

$$\begin{aligned} \log(X^i_{B(t)}) = &\log(X^i_{B(t-1)} e^{-K_B \Delta t} + X^i_{D(t-1)}(1 - e^{-K_D \Delta t})\pi_{DB} \\ &+ X^i_{R(t-1)}(1 - e^{-K_R \Delta t})\pi_{RB} + X^i_{H(t-1)}(1 - e^{-K_H \Delta t})\pi_{HB} \\ &+ X^i_{B(t-1)}(1 - e^{-K_B \Delta t})\pi_{BB}) + \eta^i_{B(t)}, \quad \eta^i_{B(t)} \sim N(0, \sigma^2_{\eta_B}). \end{aligned} \tag{24}$$

The transition density of the joint process model of the latent variables in this model can be gained through multiplying the transition densities of the latent variables in this model as they are independent.

G.2 Observation Model

The observation model of the five-pool model captures the uncertainties in the observations of the carbon input, TOC, POC, HUM, and IOM. The observation models of the carbon input and IOM which are presented by equations (9)-(13) and (17) are the same in this model. The measurement processes of the TOC, POC, and HUM given their related state

variables (e.g. $X^i_{D(t)} = x^i_{D(t)}$) are

$$\log(Y^i_{TOC(t)}) = \log(x^i_{D(t)} + x^i_{IOM(t)} + x^i_{B(t)} + x^i_{R(t)} + x^i_{H(t)}) + \eta_{\epsilon TOC}, \quad \eta_{\epsilon TOC} \sim N(0, \sigma^2_{\epsilon TOC}); \tag{25}$$

$$\log(Y^i_{POC(t)}) = \log(x^i_{D(t)} + x^i_{B(t)} + x^i_{R(t)}) + \eta_{\epsilon POC}, \quad \eta_{\epsilon POC} \sim N(0, \sigma^2_{\epsilon POC}); \tag{26}$$

$$\log(Y^i_{H(t)}) = \log(x^i_{H(t)}) + \eta_{\epsilon H}, \quad \eta_{\epsilon H} \sim N(0, \sigma^2_{\epsilon H}). \tag{27}$$

Since the measurement variables in (25)-(27) are independent, the product of the probability density functions of equations (9)-(13), (17), and (25)-(27) yields the joint observation model of the five-pool model.

H Gelman and Rubin's Convergence Diagnostic Statistic

I Estimated LPD and ELPD of the Models

The mean and standard deviation of the fourchains of the estimated LPD at each time point and the ELPD of the models applied on the Tarleeand Brigalow datasets are shown in Tables S13 and S14, respectively.

J Hardware Use and Computing Time

The codes are run on High Performance Computing (HPC) in R version 3.5.1. The elapsed running time, per minute, of the codes per 1000 MCMC iterations is provided in Table S15.

Parameter	$\hat{R}$	Upper C.I. bound on $\hat{R}$
K_C	1.00	1.00
c	1.00	1.00
r_W	1.00	1.00
r_P	1.00	1.00
p	1.00	1.00
h_W	1.00	1.01
μ_{G_W}	1.03	1.08
μ_P	1.00	1.00
ρ_{G_W}	1.06	1.16
ρ_P	1.00	1.00
$\sigma^2_{\eta_C}$	1.05	1.07
$\sigma^2_{G_W}$	1.01	1.02
σ^2_W	1.07	1.11
σ^2_P	1.01	1.01
Y_{IOM}	1.00	1.00
$\sigma^2_{\eta_B}$	1.02	1.04
K_B	1.00	1.00
π_{CB}	1.00	1.01
π_{BB}	1.00	1.00
π_{BC}	1.00	1.00

Table S11: The Gelman and Rubin's convergence diagnostic, $\hat{R}$ calculated for model parameters of the three-pool model of the Tarlee dataset. Since the point estimate of $\hat{R}$ for each parameter is less than 1.2, the MCMC samples can be considered to have reached a stationary distribution and are mixing adequately.

Parameter	$\hat{R}$	Upper C.I. bound on $\hat{R}$
K_C	1.00	1.00
c	1.00	1.00
r_W	1.00	1.00
p	1.01	1.03
h_W	1.01	1.02
μ_{G_W}	1.10	1.23
μ_{G_S}	1.00	1.00
ρ_{G_W}	1.01	1.03
σ^2_η	1.07	1.19
$\sigma^2_{G_W}$	1.00	1.00
σ^2_W	1.01	1.01
r_S	1.00	1.00
σ^2_B	1.14	1.35
σ^2_S	1.10	1.30
K_B	1.00	1.02
K_R	1.02	1.07
π_{DB}	1.00	1.00
π_{BB}	1.00	1.00
π_{BC}	1.00	1.02
π_{CB}	1.05	1.17
μ_{G_S}	1.00	1.00
ρ_{G_S}	1.00	1.00
$\sigma^2_{G_S}$	1.05	1.07
h_S	1.01	1.02
ρ_{G_S}	1.00	1.00

Table S12: The Gelman and Rubin's convergence diagnostic, $\hat{R}$ calculated for model parameters of the three-pool model of the Brigalow dataset. Since the point estimate of $\hat{R}$ for each parameter is less than 1.2, the MCMC samples can be considered to have reached a stationary distribution and are mixing adequately.

Time	One-pool model		Two-pool model		Three-pool model		Five-pool model	
	Mean (LPD)	SD (LPD)	Mean (LPD)	SD (LPD)	Mean (LPD)	SD (LPD)	Mean (LPD)	SD (LPD)
13	-5.27	0.44	-3.21	0.27	-2.40	0.04	-2.67	0.02
14	-8.14	0.41	-8.34	0.48	-7.22	0.17	-6.35	0.10
15	-5.53	0.30	-3.72	0.08	-3.26	0.04	-2.85	0.01
16	-7.82	0.41	-7.32	0.77	-6.09	0.24	-6.09	0.27
17	-6.45	0.86	-5.15	0.65	-5.34	0.36	-3.62	0.02
18	-7.70	0.50	-6.42	0.21	-5.11	0.21	-4.97	0.12
19	-5.66	0.44	-3.02	0.11	-2.59	0.03	-3.04	0.03
20	-6.45	0.44	-3.33	0.25	-2.78	0.71	-7.41	0.61
ELPD	-53.02	3.80	-40.55	2.82	**-34.79**	1.80	-37	1.18

Table S13: The mean and standard deviation (SD) of the four chains of the estimated LPD and ELPD of the SOC models applied on the Tarlee dataset.

Time	One-pool model		Two-pool model		Three-pool model		Five-pool model	
	Mean (LPD)	SD (LPD)	Mean (LPD)	SD (LPD)	Mean (LPD)	SD (LPD)	Mean (LPD)	SD (LPD)
14	-9.82	0.82	-9.93	1.70	-8.62	0.45	-9.60	1.71
15	-7.13	0.22	-6.88	0.14	-6.97	0.34	-7.40	0.43
16	-7.86	0.50	-7.96	0.87	-8.51	0.47	-8.62	0.54
17	-3.69	0.06	-3.71	0.05	-3.77	0.06	-3.94	0.03
18	-6.84	0.28	-6.74	0.05	-6.57	0.56	-7.95	0.98
19	-1.55	0.03	-1.66	0.03	-2.04	0.06	-12.06	0.89
ELPD	-36.89	1.91	-36.88	2.84	**-36.48**	1.94	-49.57	4.58

Table S14: The mean and standard deviation (SD) of the four chains of the estimated LPD and ELPD of the SOC models applied on the Brigalow dataset.

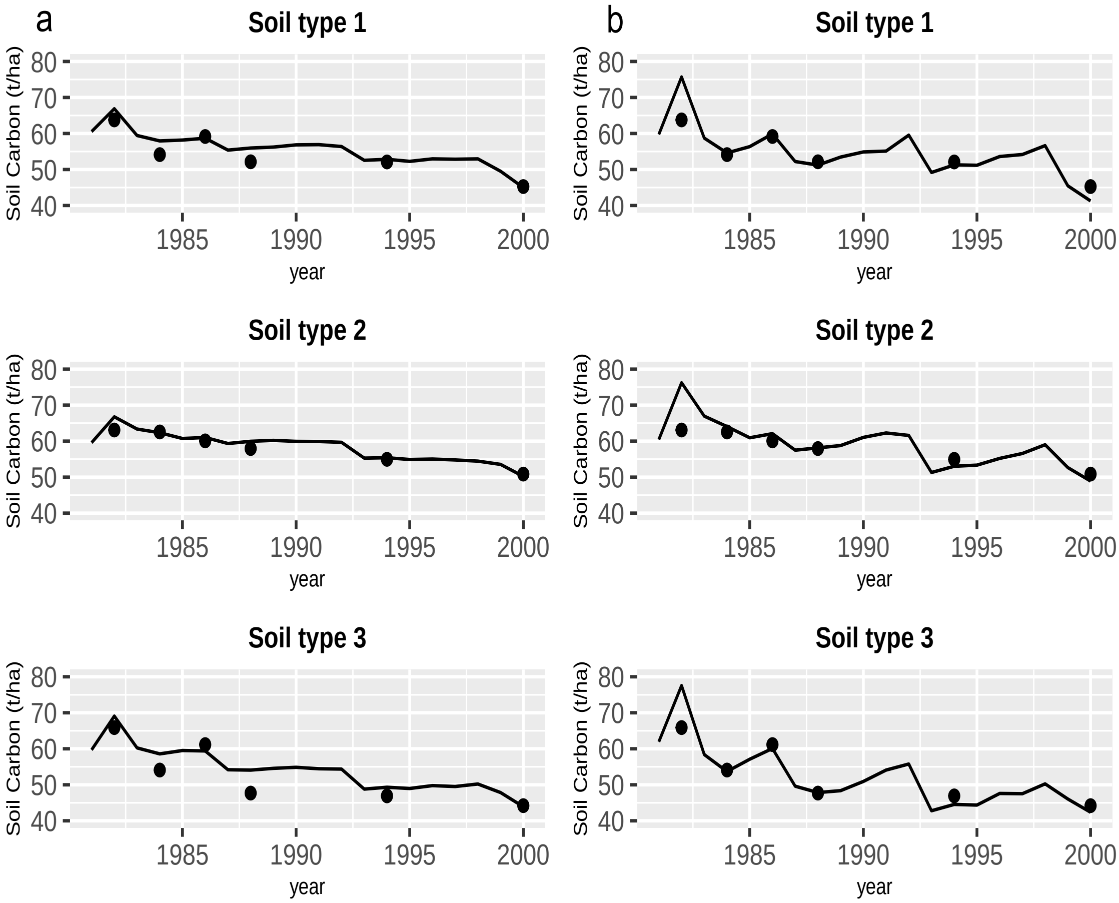

Figure S1: Soil organic carbon (SOC) dynamics of the Brigalow dataset based on a) the three-pool model and b) the five-pool model. The 50th percentile is shown by the solid line and the measured SOC values are indicated by filled dots.

Model	Time (Mins)	
	Brigalow	Tarlee
One-pool	0.83	2.20
Two-pool	1.29	3.03
Three-pool	1.46	1.53
Five-pool	1.50	2.07

Table S15: The elapsed running time (per minute) of the codes. The elapsed time is based on 1000 MCMC iterations.
